# Current Advances in Lipid Nanosystems Intended for Topical and Transdermal Drug Delivery Applications

**DOI:** 10.3390/pharmaceutics15020656

**Published:** 2023-02-15

**Authors:** Nakamwi Akombaetwa, Ange B. Ilangala, Lorraine Thom, Patrick B. Memvanga, Bwalya Angel Witika, Aristote B. Buya

**Affiliations:** 1 Faculty of Pharmacy, Nutrition and Dietetics, Lusaka Apex Medical University, Lusaka 10101, Zambia; 2 Faculty of Pharmaceutical Sciences, University of Kinshasa, Kinshasa XI B.P. 212, Democratic Republic of the Congo; 3 Interfaculty Research Centre on Biomaterials (CEIB), Chemistry Institute, University of Liège, B6C, 11 Allée du Août, 4000 Liege, Belgium; 4 Department of Pharmaceutical Sciences, School of Pharmacy, Sefako Makgatho Health Sciences University, Pretoria 0204, South Africa; 5 Centre de Recherche et d’Innovation Technologique en Environnement et en Sciences de la Santé (CRITESS), University of Kinshasa, Kinshasa XI B.P. 212, Democratic Republic of the Congo; 6 Centre de Recherche en Sciences Humaines (CRESH), Ministère de la Recherche Scientifique et Innovation Technologique, Kinshasa XI B.P. 212, Democratic Republic of the Congo

**Keywords:** lipid nanosystems, nanoparticles, dermal delivery, transdermal delivery, targeted delivery

## Abstract

Skin delivery is an exciting and challenging field. It is a promising approach for effective drug delivery due to its ease of administration, ease of handling, high flexibility, controlled release, prolonged therapeutic effect, adaptability, and many other advantages. The main associated challenge, however, is low skin permeability. The skin is a healthy barrier that serves as the body’s primary defence mechanism against foreign particles. New advances in skin delivery (both topical and transdermal) depend on overcoming the challenges associated with drug molecule permeation and skin irritation. These limitations can be overcome by employing new approaches such as lipid nanosystems. Due to their advantages (such as easy scaling, low cost, and remarkable stability) these systems have attracted interest from the scientific community. However, for a successful formulation, several factors including particle size, surface charge, components, etc. have to be understood and controlled. This review provided a brief overview of the structure of the skin as well as the different pathways of nanoparticle penetration. In addition, the main factors influencing the penetration of nanoparticles have been highlighted. Applications of lipid nanosystems for dermal and transdermal delivery, as well as regulatory aspects, were critically discussed.

## 1. Introduction

Due to recent technological advances in the field of drug delivery (e.g., the fabrication of functionalised biopolymers) and the growing demand for more localised delivery to reduce side effects, the oral route of administration is becoming less popular [1]. Skin delivery offers an attractive alternative to oral drug delivery. The skin, the first line of defence between the body and the outside world, protects the body from external aggressions [2]. The thickness of the skin on different areas of the body varies greatly, but the underlying structure remains the same. Skin delivery can be broadly classified into two types: dermal (topical) and transdermal drug delivery. On the one hand, dermal delivery refers to the delivery of a drug directly to the site of action on the surface of the skin, resulting in a higher localised concentration of the drug and reduced systemic exposure [3]. On the other hand, transdermal delivery refers to the transport of the drug from the surface of the skin into the systemic circulation to achieve therapeutic levels. For years the skin has been used for the local administration of active substances, and this route has also gained popularity for systemic delivery through the introduction of transdermal patches [4]. The main reason for the increased interest in transdermal delivery, could be the rise in chronic skin diseases, the desire for targeted delivery and enhanced patient-compliance [3,5]. Topical and transdermal delivery of a drug have considerable therapeutic potential due to the large surface area of the skin [6]. Furthermore, transdermal delivery allows controlled release of the drug into the interior regions [7] thus minimizing substantial fluctuations in plasma concentration, especially for repeated dosing [8]. The corresponding stable blood level might avoid severe side effects. One of the main advantages of transdermal administration is the avoidance of first-pass metabolism and the gastrointestinal (GI) tract, thus improving the bioavailability of the drug. This route allows comfortable and painless self-administration for patients and is a sensible way to administer drugs in cases where the patient cannot tolerate oral administration (such as paediatric, nauseous and comatose patients).

Generally, the acceptability of topical and transdermal products is extremely high, which is also evident from the growing market for these products. This is demonstrated by the fact that the value of skin delivery, which was estimated to be approximately $12.838 billion in 2020, is anticipated to rise to $13.457 billion by 2026 with a compound annual growth rate higher than that of other delivery routes [9]. Indeed, more than 36 transdermal products and 16 active pharmaceutical ingredients have been approved for clinical use in the US market [10], and some of them are presented in Table 1. Many of the transdermal medications currently marketed are indicated for pain, stenocardia, hypertension, contraception, or motion sickness. For instance, BUTRANS^®^ provides a gradual systemic delivery (7 days) of buprenorphine to reduce the pain associated with long-term disorders. CATAPRES-TTS^®^, a transdermal system that provides continuous delivery of clonidine, a central alpha-agonist used for hypertension. ESTRADERM^®^ releases oestradiol through a rate-limiting membrane, delivering the hormone weekly and thereby improving patient adherence. 

Despite the increased interest in skin delivery, the main challenge is to overcome the sophisticated barrier function of the impermeable stratum corneum, which impedes drug absorption. Many attempts have been made to weaken or destabilise skin layers to improve drug permeation. The first approach used to overcome the epidermal barrier is the use of chemical enhancers such as glycols, ethanol, terpenes, and azones [5,11]. They improve drug delivery by enhancing drug partition and partially fluidizing skin lipids. The second approach is the use of physical enhancement techniques, such as electroporation, microneedles, sonophoresis, iontophoresis, magnetophoresis, microdermabrasion and thermal ablation [10,12,13,14]. These techniques deliver the drug directly to the targeted skin layer, bypassing the stratum corneum. Both approaches have proven effective in delivering a variety of drugs. However, chemical permeation enhancers can irritate the skin and even cause irreversible skin damage, while physical techniques are usually painful and expensive and do not guarantee patient compliance [5]. The third and most famous approach is the use of drug delivery systems. These systems can increase drug solubility, drug distribution, and membrane fluidity, all of which help improve drug transport through the skin [15]. Among the many drug delivery systems that have been developed, nanoparticulate drug delivery has shown great promise for dermal and transdermal drug delivery over the past decades.

Many nanoparticulate systems have been explored for the delivery of active pharmaceutical ingredients (API) and development of cosmetics of for dermal and transdermal applications. Particularly, polymeric nanoparticles [16], metallic nanoparticles [17], nanocrystals [18], nanosponges [19] and lipidic nanocarriers have been extensively used in dermal and transdermal applications. However, lipid based nanocarriers have found more utility due to many desirable features such as small particle size, which ensures close contact with the stratum corneum, drug permeation through skin is facilitated [20] and the possibility for sustained drug release from lipid nanocarriers, which ensures prolonged drug release and optimal systemic absorption of drug [21,22]. In addition, there is a reduction in transdermal water loss due to occlusive properties of lipid nanocarriers, resulting in skin hydration, elasticity, and facilitated drug penetration [23,24] as well as their suitability for topical use on damaged or inflamed skin [25,26].

Lipid-based nanosystems are primarily composed of biocompatible and biodegradable components that provide targeted delivery, controlled release, and drug protection. More recently, the development of commercial lipid-based nanosystems, such as Lipoplatin^®^ (cisplatin), Sandimmune^®^ (cyclosporine), Myocet^®^ (doxorubicin) and Neoral^®^ (cyclosporine), has stimulated growing interest in the use of these systems. Depending on the nanosystem selected and its composition, lipid-based nanosystems can be designed to target various skin disorders. To date, there have been several studies and patents on the skin delivery of lipid-based nanosystems, which have been developed to treat various diseases locally or transdermally [27]. However, formulating these nanosystems requires a sound understanding of drug characteristics, formulation and process variables, skin delivery mechanisms, specific limitations, and recent advances in the field. In order to address these aspects, this review article focuses on lipid-based nanosystems for topical and transdermal applications with particular consideration of recent research, progress and challenges in dermal and transdermal delivery.

## 2. Skin Anatomy and Drug Penetration Pathways/Routes

### 2.1. Anatomy and Functions of the Skin

Human skin is the largest organ in the body. The average skin surface of an adult male is approximately 2 m^2^. The main functions of the skin are prevention of water loss, protection against mechanical stress, cooling by perspiration, and prevention of foreign body absorption [28]. From the outside in, the skin has three layers: the epidermis, the dermis, and the hypodermis, also called the subcutaneous tissue [29]. Each layer has a function and is an important part of the skin.

The epidermis is a thin stratified layer of epithelium that develops from the ectoderm and acts as a barrier (physical and chemical) between the inside of the body and the outside environment [30]. It is composed of melanocytes, Merkel cells, and Langerhans cells that rest on a dermis containing the nervous and vascular networks [31,32]. Since the epidermis does not contain blood vessels, molecules that penetrate the epidermis must cross the dermo-epidermal layer before entering the body’s systemic circulation. It is completely nourished by the underlying dermis and eliminates waste by diffusion through the dermoepidermal junctions and the surface of the skin. It is divided into two regions: the nonviable and viable epidermis. The nonviable epidermis contains 70% water and keratinizing epithelial cells, which are responsible for the synthesis of the stratum corneum, the outermost skin layer, involved in homeostatic and protective functions [28]. The stratum corneum, which is approximately 10–20 µm thick and is considered metabolically inactive, is the product of epidermal differentiation. It consists of 15 to 30 layers of dead, enlarged, totally keratinized corneocytes enclosed in a lipid matrix [33]. It generally resembles a mortar and brick structure, with the hydrated keratinous corneocytes of the skin resembling bricks embedded in a mortar containing extracellular lipid components. Extracellular lipids are composed of two lamellar phases: a predominant crystalline phase and a subpopulation in the liquid lipid phase [34]. The viable epidermis is approximately 50–100 µm thick and lies beneath the stratum corneum [35]. It differs from the stratum corneum in that it is physiologically closer to other living cellular tissues and has a greater number of enzymes that aid in metabolism. The epidermis is responsible for producing the corneal layer and degrading foreign compounds. Langerhans cells are also involved in the skin’s immune response [33,36].

The dermis is a layer of connective tissue that exists between the epidermis and the subcutaneous tissue. It is composed of proteins (collagen), elastic tissue, interfibrillar gel of glycosaminoglycans, salt, and water [37]. Other extracellular components embedded in the dermis include blood and lymphatic vessels, hair follicles, nerve endings, and glands (sebaceous and sweat [38]. The dermis supports, protects, and nourishes the skin while helping with thermoregulation and sensations. In addition to fibroblasts, the dermis also contains adipocytes, mast cells, and histiocytes, all of which help maintain its structure and function.

### 2.2. Pathways for Skin Penetration

**Figure 1:**
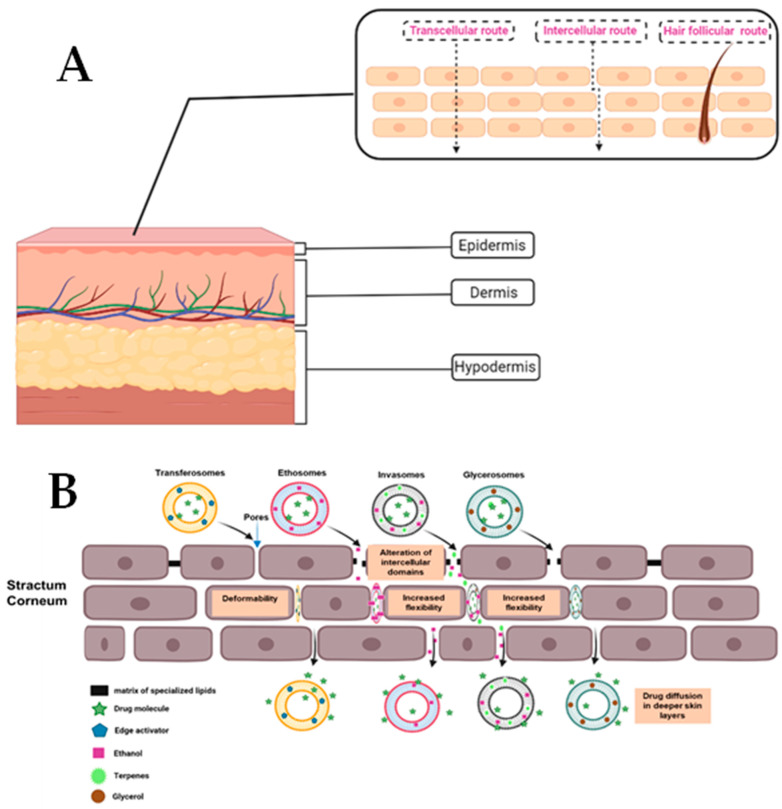
Graphic illustration of the structure of the skin and pathways for skin penetration of nanoparticles (**A**) and the mechanisms involved in advanced vesicular skin penetration (**B**). Adapted with permission from [54] and Elsevier B.V Netherlands.

The skin is not a perfect barrier and does not completely prevent the absorption of topical substances. The three possible routes of penetration of a compound applied topically through the skin are well known: (i) the hair follicle route; (ii) the intracellular route; and (iii) the intracellular route (Figure 1). The importance of these pathways for the transcutaneous absorption of substances is determined by the length and frequency of their paths, as well as the solubility and diffusivity of the compound in each domain [39].

#### 2.2.1. The Shunt Route

Hair follicles, which are connected to systemic blood circulation at their base, can serve as a channel from the epidermis to the dermis [40,41]. Sweat glands or hair follicles create a "shunt" pathway that allows penetrant to pass via the stratum corneum. It has already been demonstrated that nanoparticles penetrate deeper into the follicles than small free molecules [42]. This has prompted the development and delivery of nanosystems to hair follicles, where they could accumulate drugs and efficiently release them into the systemic bloodstream. Targeting specific cells, such as epithelial stem cells and melanocytes involved in regeneration and pigmentation, may be possible by delivering particles through hair follicles. Of course, particle size has a significant impact on penetration [43]. Ladenman et al. demonstrated that nanoparticles with a size of 320 nm had significantly greater penetration into hair follicles than nonparticulate substances of the same molecular size [44,45]. Additionally, it has been shown that hair follicles can store drugs for a long time and allow them to enter the body faster than other pathways [43]. This suggests that some submicron particles can penetrate and accumulate more efficiently than larger particles. According to this theory, the physical characteristics of the particle have a greater influence on follicular penetration than the chemical characteristics. There is still a need for systematic data on other physical factors that may influence follicular penetration, such as particle surface shape and texture [46,47,48,49].

#### 2.2.2. Intracellular Route

The intracellular (transcellular) route involves permeation through corneocytes followed by intercellular lipids. Compounds entering through this pathway take advantage of imperfections in the corneocytes, which create water-filled openings [50]. The chemical moves through the corneocytes, which have separated from the cell membrane and are filled with keratin. This pathway is not regarded as the preferred route for dermal diffusion due to the extremely low permeability of corneocytes and the need to repeatedly separate the more hydrophilic corneocytes from the lipid intercellular layers of the stratum corneum and vice versa [51]. The intracellular route may become more important when a penetration enhancer is used because it may alter the protein structure of corneocytes and therefore their permeability.

#### 2.2.3. Intercellular Route

The intercellular pathway is the main entry route for most substances, especially when steady-state conditions have been reached. Solutes move through intercellular lipid domains by diffusing through horny cells in the stratum corneum, viable cells in the epidermis, and the dermis [51]. According to tracer studies, intercellular lipids rather than corneocyte proteins are the main epidermal permeability barriers [52]. Due to its low volume of occupancy, the intercellular pathway was later dismissed as the dominant skin permeation mechanism [30]. However, it was later found that the intercellular volume fraction was much larger than originally estimated [53]. These results suggest that the intercellular pathway provides a significant barrier to skin permeation.

A summary of the skin anatomy and how it relates to the routes of skin penetration by nanomaterials as well as the mechanisms of penetration is provided in Figure 1.

## 3. Lipid Nanosystems Utilized in Skin Delivery

Achieving the desired response to therapeutic agents in a wide range of disease states largely depends on direct delivery of the drug to a specific site [55]. However, the delivery of these therapeutic agents is limited by numerous biological barriers. Biological barriers that limit drug delivery to desired sites at required rates include the skin, mucosal membranes, the blood–brain barrier and cell and nuclear membranes. The general barrier function of these anatomical structures serves as a means of protecting the body from invading pathogens; thus, drug delivery becomes limited [56]. Topical drug delivery is characterised by low drug efficacy because of poor skin penetration or skin permeation of drugs from the most conventional formulations. Shifting the penetration pathway of drugs from transcellular to intercellular or follicular is one way of bypassing the stratum corneum of the epidermis, which is the major skin barrier [57]. One of the most used approaches to overcome these barriers and improve therapeutic outcomes is the use of lipid nanosystems. These nanosystems involve the use of advanced excipients, carriers, or permeation enhancers to increase drug permeation [56]. Lipid nanosystems are carriers of choice for topically applied drugs and include lipid-based nanoparticles and vesicular systems [58,59]. The small size of these carriers eases drug permeation through the skin; they provide for modified drug release; their occlusive effect on the skin results in skin hydration and elasticity that facilitates drug permeation; and due to their non-irritant and nontoxic properties, they are safe to use on damaged or inflamed skin [22].

### 3.1. Classification of Lipid Nanosystems

Lipid nanosystems come in two basic morphologies viz., particulate and vesicular. The main purpose of particulate shape systems is to solubilise hydrophobic compounds within their lipid matrix. In contrast, vesicular carriers are primarily intended to deliver hydrophilic drugs, but a small number of hydrophobic drugs can be incorporated into the bilayer [60]. They are also classified based on their structural composition, which influences not only the types of drugs they can carry, but also their inherent drug loading capacity as well as modified drug release and/or targeted drug delivery in deeper skin layers where the drugs are most useful and can better fulfil their pharmacological activity [58,61,62].

**Figure 2:**
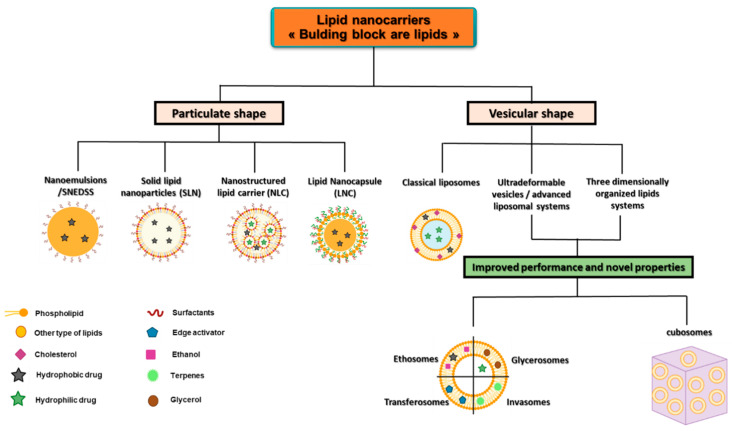
Classification of the main types of lipidic nanosystems for dermal and transdermal drug delivery. Adapted with permission from [62] and Elsevier B.V Amsterdam.

Since their successful introduction in the cosmetic and pharmaceutical fields over 30 years ago, their chemical compositions and structural features of nano systems have significantly evolved. Currently, different formulation strategies are proposed, ranging from classical delivery systems such as liposomes to novel lipid-based nanostructures with improved/superior delivery properties capable of addressing many limitations encountered with conventional nanosystems. A summary of particulate and vesicular lipid nanosystems is provided in Figure 2.

#### 3.1.1. Particulate Carrier Systems

##### Nanoemulsions

Nanoemulsions are metastable dispersions of two immiscible liquids consisting of oil, surfactant, cosurfactant, and water in appropriate ratios [63]. They are thermodynamically stable systems with an extremely small droplet size ranging from 20 to 200 nm and therefore display different physicochemical and biological properties than traditional emulsions (>500 nm) [64]. Nanoemulsions exist in three distinct forms: (i) oil in water (O/W), wherein oil droplets are dispersed in a continuous aqueous phase, (ii) water in oil (W/O), wherein water phases are dispersed in a continuous oil phase, and (iii) bicontinuous/multiple emulsions, where microdomains of oil and water phases are interdispersed within the system [65]. The oil phase plays a vital role in nanoemulsion formulation, as it helps solubilise lipophilic drugs, thereby achieving higher encapsulation rates of poorly soluble drugs than conventional gels, creams, and lotions [66]. Nevertheless, hydrophilic drugs, including large therapeutic modalities such as proteins and oligonucleotides, could be effectively loaded into multiple water-in-oil-in-water (W/O/W) emulsions [67,68,69]. The composition of colloidal dispersions dictates their physicochemical properties and the way they interact with the skin. During the formulation development of nanoemulsions, a prudent selection of the most promising emulsifier systems based on their solubility and emulsification ability has to be made using a pseudoternary phase diagram that is constructed from different formulation components [69]. In addition to phase diagrams, a quality by design approach is also recommended to systematically refine the desirable physicochemical properties of nanoemulsion formulations in order to meet specific drug delivery requirements, either for topical (promote drug deposition in epidermis and dermis layers of the skin) or for transdermal applications (effective flux of drug through skin into blood circulation) [70,71].

Although several mechanisms have been suggested to explain the effect of nanoemulsions on skin permeation in the literature, definitive answers to this attractive question are still not yet fully provided. Considerable research is still conducted using various skin models to aid the understanding of this complex process [72,73,74]. Basically, both drug and carrier-related parameters have an impact on skin penetration. The following are the most commonly accepted arguments put forward to elucidate the mechanism of enhanced skin penetration of nanoemulsions: (i) increases drug thermodynamic activity, thereby resulting in better drug portioning into the skin, (ii) modification of the surface electrical charge of ionic drugs, (iii) modification of the diffusional barrier of the skin by solubilisation of sebum and hydration of the stratum corneum layer to favour better interaction of hydrophilic drugs with epidermal and dermal cells, and (iv) promotion of pore pathways and follicular delivery of drugs due to the small and narrow size distribution of droplets.

##### Solid Lipid Nanoparticles (SLNs) and Nanostructured Lipid Carriers (NLCs)

In these carrier systems, the drug is either dissolved or molecularly dispersed in the solid lipid matrix that restricts the mobility of the entrapped drug, thereby providing a controlled and sustained release profile. SLNs, developed in early 1990, are submicron colloidal carriers ranging from 50 to 1000 nm, which are composed of physiological lipids dispersed in water or in aqueous surfactant solution [75]. A wide range of lipids that are solid and stable at room temperature (i.e., triglycerides, free fatty acids, and free fatty alcohols) have been used to produce SLNs. Most of those lipids are physiological, inexpensive, and nontoxic, which make SLNs a widely promising platform for topical drug delivery. Depending upon the type of employed lipids, critical SLN physicochemical properties will vary, including particle size, drug entrapment efficiency, and drug release kinetics. SLN formulations are typically composed of solid lipids (0.1–30%), water, and emulsifiers (0.5–5% *w*/*w*) [76]. There are various techniques to produce SLNs, including easily scaled-up and organic solvent-free techniques such as high-pressure homogenisation (HPH) [77]. Numerous scientific publications are reporting successful implementation of different quality by design strategies for SLN-based product development, bringing additional benefits to pharmaceutical manufacturers [78,79]. Other advantages of SLNs for skin delivery include good UV-blocking property, improvement of skin hydration, enhanced protection of the encapsulated drug, and possible application to damaged skin [80]. However, SLNs have some limitations, such as low drug loading capacity and uncontrolled expulsion of the entrapped drug from SLNs as a result of polymorphic transitions and crystallisation of the lipid matrix that occur during storage [58]. To overcome these limitations, the second generation of lipid nanoparticles, known as nanostructured lipid carriers (NLCs), has emerged. The NLC matrix is made up of both solid and liquid lipids organised in a spatially different way. The introduction of liquid lipids in the solid matrix creates imperfections in the crystal lattice of nanoparticles, leading to increased drug loading capacity and solubility, and lower leaching properties compared to SLNs. The solid and liquid lipids are typically blended in the ratio of 70/30 up to 99.9/0.1, and the amount of surfactant ranges from 1.5% to 5% (*w*/*v*) [81].

Enhanced skin penetration of SLNs and NLCs is described to be a combination of three mechanisms, namely, adhesiveness, occlusiveness, and skin hydration. Adhesiveness is related to SLN/NLC physiological lipid composition that triggers interaction with the stratum corneum, impacting its lipid reorganisation, which enhances molecule penetration. The small size of the nanoparticles also contributes to increasing their adhesiveness and surface contact area [82]. The occlusiveness effect is dependent on the concentration of lipids, sample volume applied, particle size, and crystallinity of particles [83]. The succession of the above properties contributes to prevention of transepidermal water loss and opening of intergaps between corneocytes, promoting penetration of therapeutic agents deep into skin layers [84].

##### Lipid Nanocapsules (LNCs)

Lipid nanocapsules (LNCs) are a versatile carrier system with a size between 20 and 100 nm and are widely used in drug delivery applications with different routes of administration. LNCs consists of a lipid core, generally medium-chain triglycerides (caprylic/capric triglycerides) surrounded by capsules containing hydrophilic and lipophilic surfactants, such as polyethylene glycol (PEG)-derivative surfactants and lecithin, respectively [85]. The properties of LNCs can be modulated greatly by altering their composition, and they can be tailored to accommodate a broad range of hydrophobic and hydrophilic APIs [86]. They are therefore considered as a promising alternative carrier to conventional emulsions and polymeric nanoparticles. LNCs are prepared by a phase inversion method, which is a solvent-free method based on thermal manipulation of an oil/water system.

In recent years, the rationale of utilising LNCs for dermal and transdermal drug delivery has been successfully evaluated due to the unusual colloidal characteristics of these carriers and the excellent tolerability of FDA approved materials involved in their preparation. They also offer additional benefits, such as long-term stability under storage and protection of encapsulated compounds.

Literature focusing on the mechanistic aspects of NLC interactions with the skin is hardly available, although numerous investigations performed mainly by confocal laser scanning microscopy using fluorescently labeled LNC formulations have demonstrated that LNCs preferentially accumulate in the stratum corneum and even reach the viable epidermis. A study conducted by Zhai et al. [87] suggested that LNCs promote skin penetration by breaking the close conjugation of corneocyte layers. However, many researchers consider LNCs to have limited potential for the transdermal delivery of APIs.

#### 3.1.2. Vesicular Carrier Systems

##### Conventional Liposomes

Over the past few decades, research on liposomal drug delivery systems has expanded significantly. Currently, liposomes (LPs) represent the most investigated and clincally established lipid nanosystems with a variety of medical applications, including topical and transdermal drug delivery [88,89]. LPs are spherical vesicular lipid-based nanocarriers consisting of phospholipids and cholesterol, the main building blocks of biological membranes, explaining their remarkable safety and biocompatible profiles. Their typical bilayer structure surrounding an aqueous compartment has made them a suitable carrier for molecules with opposing solubility, hydrophilic and hydrophobic active compounds [90]. The exceptional expansion in medical applications of liposomes comes as a result of progress recorded in the lipid chemistry field. Indeed, a vast option of phospholipids with attractive functionalities has been made available over the last decade. For research and development prospects, this simply means that one can currently design liposomes with fine-tuned physicochemical characteristics by a careful selection of lipids having desirable properties in terms of phase transition behaviour, hydrophilicity, hydrophobicity, charge, fusogenic, and stimuli-responsive properties [91,92,93,94]. All these factors help modulate the interactions between liposomal systems and biological systems to unlock their maximum therapeutic potential (high drug loading capacity, optimal drug release, intracellular uptake and trafficking ability in different skin layers). Considerable advancement and innovation have also been made in the manufacturing of liposomes, from laboratory scale to large scale production, speeding up the adoption and clinical translation of new liposomal systems [95]. Regarding topical and transdermal drug delivery, phospholipids, a key structural element of liposomal carriers, easily combine with skin lipids and ensure ideal moisture conditions for enhanced drug penetration and localisation in the layers of the skin [96]. Additionally, they have shown a tremendous improvement in the therapeutic index and a significant reduction in the adverse effects related to topically used drugs, such as anti-inflammatory and antipsoriatic medications [97,98].

Despite much investigation into the mechanism of transport of drugs loaded into liposomes, it remains a much-debated concept. Liposomes offer enhanced skin penetration by the following mechanisms: (i) intact vesicular skin permeation, (ii) acting as a penetration enhancer, inducing changes in the stratum corneum’s constituents, structure, and thermodynamic characteristics, (iii) adsorption of liposomes made of skin-like lipids such as ceramides and free fatty acids and (iv) enhanced transport through the transappendageal route guided by particle size of liposomes [99,100,101,102,103,104,105,106,107].

##### Ultradeformable Vesicles and Advanced Liposomal Systems

As described in previous sections, nanosystems can be transported through the epidermis via three distinct routes: intercellular, transcellular, and follicular routes. However, many researchers still view the intercellular pathway as holding enormous delivery potential that remains underexplored using conventional vesicular systems. Thus, driven by the need to further enhance the skin penetration efficiency of conventional vesicles (i.e., classical liposomes), which rarely provide satisfactory transdermal effects, new classes of ultradeformable vesicles have been designed by structural and chemical modifications of liposomes [54,108,109]. These relatively new vesicular systems, due to their smaller size and, more importantly, their ability to press through intercellular lipid bilayers present in the stratum corneum, exhibit superior delivery of cargo drugs to deeper skin layers (dermis), from which they can be absorbed into the bloodstream [110,111]. They are further classified based on their composition.

Transferosomes are the first generation of ultradeformable vesicles introduced by Cevc and Blume, and they contain, in addition to phospholipids, other substances in their membranes, such as single-chain surfactants [112]. These substances, also known as “edge activators”, improve the deformability of the bilayers primarily by varying their interfacial tension. Sodium deoxycholate, Span^®^ 60, Span^®^ 65, and Span^®^ 80 are the most commonly used edge activators in the preparation of transferosomes [59]. Due to their enhanced elasticity over conventional liposomes, transferosomes can penetrate intact skin when applied under nonocclusive conditions and are therefore widely considered for the noninvasive or needle-free delivery of various therapeutic agents [113,114]. It was proven that particles with a size of up to 200–300 nm can easily penetrate [114,115]. A standard formulation of transferosomes is composed of phospholipids (soy phosphatidylcholine, egg phosphatidylcholine, etc.) as the main component, single chain surfactant in a range of 10–25%, alcohol as solvent (3–10%), and phosphate buffered saline (pH 6.5–7) [116]. For the latest applications, transferosomes have been successfully used for the transdermal delivery of both low- and high-molecular-weight drugs such as insulin [117], pioglitazone and eprosartan mesylate codelivery [118], minoxidil [118], vancomycin hydrochloride [119] and tapentadol [113].

Ethosomes are recently developed novel lipidic vesicles similar to liposomes, with the only difference being that they contain a high concentration of ethanol (20–50%) in contact with the phospholipid bilayer backbone, which gives fluidity, malleability, and elasticity to the entire carrier system [120]. Regarding transdermal drug delivery, ethosomes have proven to be more effective than traditional liposomes due to their smaller size, negative zeta potential, and better entrapment efficiency for both hydrophobic and hydrophilic compounds. These characteristics are due to the presence of ethanol, a powerful penetration enhancer, which affects the average size, zeta potential, stability, drug entrapment efficiency, deeper drug penetration, and increased transdermal flux through the skin both in occlusive and nonocclusive conditions. Additional advantages include protection of sensitive drugs in the core of lipids along with the delivery of high molecular weight molecules such as protein and plasmids at the deep skin layer and/or systemic circulation [121,122]. Ethosomes range in size from 30 nm to a few microns and can be unilamellar or multilamellar in shape. Phospholipids such as phosphatidylcholine, phosphatidylethanolamine, phosphatidylserine, and phosphatic acid can be utilised to formulate ethosomes. [120,123].

Glycerosomes and invasomes are two further types of advanced liposomal systems that have drawn attention recently for the development of safe, effective, stable, and biocompatible delivery platforms meant for topical and transdermal applications. Glycerosomes are vesicular nanostructures made up of phospholipids, water, and glycerol [124]. These new vesicular systems have superior properties to conventional liposomes, including higher encapsulation efficiency, excellent permeation capability, membrane fluidity potential, and longer shelf life [124]. Although mainly described in the literature for their ability to enhance topical delivery, glycerosomes also represent one of the ideal noninvasive alternative means to improve the delivery of fragile therapeutics that cannot be administered orally due to the harsh conditions of the GI tract. Glycerol serves as an edge activator and penetration enhancer in these formulations, and increasing its content by 10, 20, or 30% results in a considerable improvement in glycerosome physical stability [125]. Several therapeutic areas including skin ailments and inflammatory and infectious diseases have recently benefited from these glycerosomes [126,127,128].

Invasomes exhibit similar delivery characteristics to other upgraded liposomal systems and have been extensively evaluated for various drug delivery outcomes through the skin [109,129]. They are composed of phospholipids, low concentrations of ethanol (3% *v*/*v*), terpenes (1–5%), and water. Dug penetration enhancement with invasomes appeared to be a combined effect of ethanol, terpenes, and the entire invasome system itself [129]. 

##### Three Dimensionally Organised Lipid Systems

Since the first study case reported in the early 90s, the family of structurally adaptable nanoparticles enveloping internally inverse non-lamellar liquid crystalline phases has been an object of great interest for several drug delivery applications [130]. These nano-self assemblies share common features with SLNs with the difference that the solid crystalline internal architecture is replaced by non-lamellar liquid crystalline phases organised in three-dimensional (3D) inverse bicontinuous cubic phase (cubosomes) or inversed hexagonal phase (hexasomes).

Although numerous amphiphilic lipids can be employed to formulate cubosomes and hexasomes, monolein-based aggregates are most frequently used in medical applications because of their GRAS status, biocompatibility, and biodegradability profile. Indeed, Monoolein (MO) or glycerol mono-oleate (GMO) can produce various cubic phase structures by simply changing the water content in either a binary GMO/water system, or a ternary one by the addition of stabilising agent, all of them subjected to high-energy emulsification methods that include ultrasonication, high-pressure homogenisation, and microfluidisation [131]. An amphiphilic non-ionic triblock copolymer of poly (ethylene oxide) (PEO) and poly (propylene oxide) (PPO), known as poloxamers, are the most used stabilisers. Moreover, depending on the purity of the sample, GMO can also form a reverse hexagonal phase in water above 80 °C [132].

However, due to its bioadhesion capabilities, the cubic phase has drawn considerable attention for topical and transdermal drug delivery [133,134,135,136]. Compared to their lamellar counterparts, liposomes, at the same size, cubosomes have a higher hydrophobic volume. They provide thereby more room to load the highest concentration of hydrophobic drugs [137]. Some additional advantages provided by these versatile nanocarriers include protection of the loaded drug from chemical and/or physiological degradation in vivo, display of drug release in a time and/or specially controlled manner due to highly organised internal structure, enhancement of bioavailability of diverse active molecules (hydrophilic, hydrophobic, amphiphilic, macromolecular drugs, etc.), and reduction of side effects observed after their administration.

The main components of cubosomes, namely glyceryl monooleate (GMO) and poloxamer are well-established penetration enhancers. GMO improves skin permeability for efficient drug delivery by encouraging the extraction of ceramide and enhancing the lipid fluidity in the stratum corneum [138]. Poloxamer, owning to its water-soluble ethylene oxide and lipophilic propylene oxide moieties, causes cubosomes to traffic more deeply into the stratum corneum [131]. Additionally, cubosomes and skin share a similar morphological organisation that makes it easier for them to press through the stratum corneum’s porous features, increasing transdermal penetration [139].

A brief overview of the lipid based nanosystems is provided in Table 2.

### 3.2. Factors Affecting Skin Penetration of Nanoparticles

There are multiple studies that have demonstrated the mechanisms with which different nanosystems achieve optimal drug delivery. The most commonly observed mechanisms involve deformation, alteration of intercellular domains and increased flexibility [54].

Furthermore, the potential ability of nanosystems to penetrate the barrier function of the skin have been investigated and illustrated [175,176]. However, there is controversy regarding the characteristics of nanosystems that affect their skin penetration. Various results were obtained using lipid nanosystems, which were attributed to the different experimental conditions in terms of exposure time, application site, skin surface, skin type and applied concentration [177]. It is essential to understand the skin permeability of lipid nanosystems and their behaviour in the different skin layers.

The critical quality attributes (CQA) of nanoparticulates that influence their performance include particle size, particle size distribution, particle shape and surface chemistry. Furthermore, the critical material attributes (CMA) of the technology have a huge impact on the performance of the lipidic technologies. The effects of these are discussed herein.

#### 3.2.1. Size

**Figure 3:**
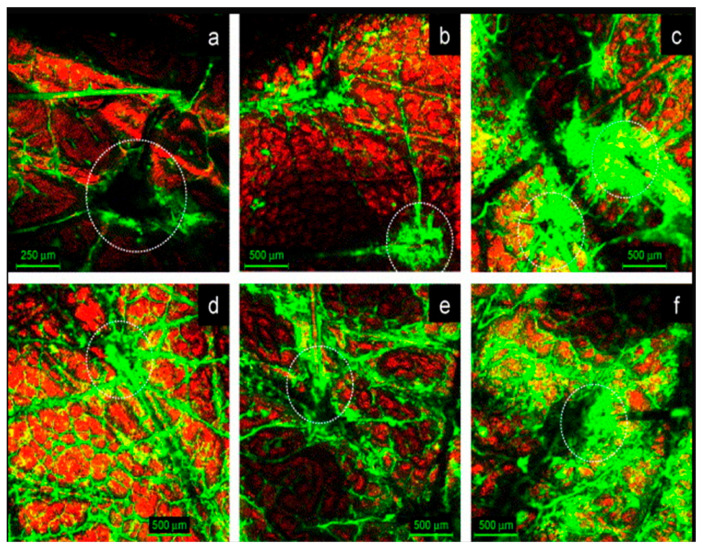
x-y images of the follicular localisation of fluorescent nanoparticles after application of nanoparticles (20 nm) for (**a**) 30 min, (**b**) 1 h and (**c**) 2 h and of nanoparticles (200 nm) for (**d**) 30 min, (**e**) 1 h and (**f**) 2 h. Hair follicles are represented by white circles. Reproduced from [184] with permission from Elsevier B.V Amsterdam. **Key:** Green fluorescence levels are localized to the follicular regions in figures (**a**–**c**), and the time-dependent accumulation of nanoparticles in hair follicles is shown in figures (**d**–**f**).

The relationship between the size of drug delivery systems and their skin penetration ability is well documented [178,179]. In general, nanosystems with a diameter of 600 nm or greater are unable to deliver encapsulated drugs to deeper skin layers [178,179]. These vesicles tend to remain on or in the stratum corneum, where they can form a lipid layer after drying. Nanovesicles with a diameter of 300 nm or less could deliver their content into the deep layers of the skin and penetrate preferentially via the transfollicular route [178]. As aforementioned in the previous sections, hair follicles serve as long-term reservoirs for topically applied drug substances, storing them for up to 10 days, which is ten times longer than the reservoir of the stratum corneum [47]. Abddel Mottaleb et al. reported results indicating that the follicular transport of nanosystems increases following a decrease in size [180]. Nanosystems with a diameter of 100 nm or less are suitable for deep skin penetration and allow the onset of action [181]. Nanoparticles smaller than 7 nm can be absorbed via lipid transepidermal pathways, while those smaller than 36 nm can be absorbed via aqueous pores [182]. Nanoparticles with diameters ranging from 10 to 210 nm, however, can preferentially pass through the transfollicular route [183]. Alvarez-Roman et al. studied the distribution of fluorescent nanoparticles of two different sizes (20 and 200 nm) on pig skin using confocal laser scanning microscopy as depicted in Figure 3. They demonstrated that the nanoparticles penetrated preferentially through the follicular pathway, facilitated by their smaller size [184].

In general, smaller particle size has a positive effect on the transdermal penetration of a drug encapsulated into lipid nanosystems. A plausible explanation for the improved skin penetration could be that the smaller the particle size is, the greater the interfacial area, which improves the drug permeability [185]. However, it is not a universal rule that a smaller globule size of a dispersion will result in increased skin penetration. Patzelt et al. showed that reductions in particle size (643 nm, 470 nm, 300 nm, 122 nm) led to a significant reduction in particle penetration depth [48].

#### 3.2.2. Shape

In particular, the shape of a nanoparticle appears to be of great importance. Disc-shaped, cylindrical, and hemispherical particles may be more successful than spherical particles at avoiding uptake by phagocytic cells [177]. They may also have improved abilities to traverse capillaries and adhere to blood vessel walls. The nature of the interaction between a nanoparticle and a lipid bilayer is highly influenced by the particle’s shape, volume, contact surface, local curvature at the point of contact, and initial orientation. This will directly impact their capacity to permeate the lipid bilayer. Mathematical studies show that, depending on how it hits the membrane, an ellipsoid particle can go through a lipid bilayer up to five times faster if it hits it in a certain way [177].

#### 3.2.3. Surface Chemistry

There appears to be a direct correlation between the surface of a nanomaterial and the performance of the technology overall. The characteristics of the surface related to parameters such as smoothness or charge have an important role and are discussed herein.

##### Surface Charge

Drug permeation can be influenced by the surface properties of nanoparticles, particularly surface charge, which is usually measured as the zeta potential. This factor is crucial in defining the in vivo behaviour of nanosystems after transdermal administration. However, there is little knowledge on how surface charges influence drug penetration, particularly regarding skin delivery. How charged nanoparticles interact with the skin has been the subject of several recent studies. Normal skin has a net negative charge, which can lead to interactions with various substances [186,187]. It has also been suggested that the polarity of lipid nanosystems and sebum lipids should be matched to maximise drug penetration. Liposomes can leave a phospholipid film on the skin, which can then interact with sebum to promote follicular drug delivery. The surface of lipid nanosystems is likely to have a positive charge to help with targeting. However, because some skin components have a negative charge, this targeting may also be linked to the skin.

Ryman-Rasmussen et al. [187] showed that nanoparticles of different surface charges can penetrate intact skin at an appropriate dose. In another study, Abdel-Mottaleb et al. [188] investigated the behaviour of different surface charged nanoparticles for selective drug delivery to inflamed skin. Compared to neutral and cationic charges, they demonstrated that surface charge had a significant impact on the tendency for penetration and accumulation in inflamed skin. It was therefore concluded that the presence of charge could enhance skin adhesion and the interaction of nanosystems, leading to a higher therapeutic effect on inflamed skin.

Recently, antigens have been encapsulated in transferosomes with opposite surface charges and inserted into dissolving microneedles to study their effects on transdermal immunisation. The results showed that cationic nanovaccines can escape endocytic compartments, allowing antigen processing via an MHC-I presentation pathway and increasing lymph node accumulation [186].

##### Hydrophilicity and Hydrophobicity

The hydrophilicity and hydrophobicity of nanoparticles results in different interactions between the nanomaterials and the cells through different mechanisms. Molecular dynamics simulations suggested that semi-hydrophilic particles can be adsorbed on the surface of the membrane bilayer, while hydrophobic nanoparticles penetrate the lipophilic core of cell membranes [189]. Indirectly, these results showed that endocytosis is the main route of uptake of semi-hydrophilic nanoparticles by cells [190]. Several studies have shown that the surface hydrophobicity of nanoparticles influences not only their penetration, but also their biodistribution and cellular uptake [191,192,193,194,195]. The circulating half-life of liposomes has been shown to increase with increasing lipid dose [196,197]. This effect is probably the result of a decrease in the phagocytic capacity of macrophages following the use of high doses of lipids or saturation of the opsonisation of circulating liposomes. Additionally, a higher degree of protein coverage was observed on hydrophobic nanoparticles compared to hydrophilic nanoparticles of comparable size [198,199].

Yang et al. formulated a hydrophobically modified nanoemulsion to improve the transdermal penetration and stability of retinyl propionate. The prepared nanoemulsions demonstrated highly enhanced transdermal drug delivery to the epidermis and dermis compared to conventional emulsions [200].

#### 3.2.4. Material Attributes

##### Oil

Oil (liquid lipid) is one of the constituents of the lipid matrix of some nanosystems such as nanoemulsions, self-emulsifying systems, SLNs and NLCs. Aside from the amount of oil use, another aspect that influence the ability of the developed system to permeate the skin is the type of oil used. Various fatty acids contained in oils have been proposed as excellent skin penetration enhancers due to their ability to interact with stratum corneum lipids [201,202]. The effect of these components on improving the penetration of lipid nanosystems is notably influenced by their chemical structure, the length of the alkyl chain, and the degree of saturation [203]. The oils with unsaturated fatty acids, when compared to saturated fatty acids of the same chain length, have been shown to result in a greater degree of skin penetration than their saturated counterparts. This has been attributed to the increased disorganizing ability of branched chain unsaturated fatty acids, resulting in the predominance of lipid disruption [204,205,206]. However, synergistic effects can be achieved by combining different oils with permeation-enhancing abilities [207,208].

##### Solid Lipid

Solid lipids constitute all or part of the lipid matrix of some lipid nanosystems, like SLNs. The type and amount of solid lipids also affect the skin penetration of the nanosystems. Using solid lipids with a low melting point and high surface activity increases skin penetration, as this can produce a narrow particle size and disrupt skin lipids. Compared to other solid lipids, Gelucire^®^ 43/01 (a mixture of mono-, di- and triglycerides with PEG esters of fatty acids) produces smaller particles, while glyceryl monostearate has excellent self-emulsifiers [209]. Due to its low melting point and reduced toxicity, acetyl palmitate is also one of the most common solid lipids used [210].

##### Non-Ionic Surfactant

Surfactants are essential for the stability and penetration of lipid nanosystems through the skin. Most of them function as penetration enhancers and have excellent ability to improve skin-nanosystems contact. Furthermore, they increase the drug’s solubility in the formulation, which increases thermodynamic stability. As a result, the type and amount of surfactant used can have a considerable impact on the skin penetration of lipid nanosystems. Non-ionic surfactants have been shown to have steric hindrance effects, which improve formulation physical stability [211]. This effect indirectly promotes absorption, which would be impeded by a larger particle size [212]. Balakrishnan et al. studied the impact of different non-ionic surfactants on skin deposition and the particle size of niosomes. The results showed that Span^®^ 20 and Span^®^ 40 (low phase transition temperature surfactants) produced higher skin deposition and smaller vesicles (214–252 nm) compared to Span^®^ 60 and Brij^®^ 52 (high phase transition temperatures) (1160–1240 nm)-containing vesicles [213].

The lipophilic surfactant on the surface of the nanosystems can act as a lubricant, thereby enhancing skin absorption. The lipophilicity of both the surfactant and formulation affects transdermal absorption [214,215]. However, although necessary, the lipophilicity of a surfactant is not always correlated with transdermal absorption. Polyoxyethylated non-ionic surfactants with a hydrophilic-lipophilic balance (HLB) value of between 7–9 have shown to be effective agents of ibuprofen skin flux [216].

Myoung and Choi investigated the transdermal penetration of isosorbide dinitrate using various non-ionic surfactants and found that those with HLB close to 7 exhibited more efficient skin permeation than higher lipophilic surfactants [217]. Furuishi et al. demonstrated that HLB values close to 8 are optimal for the transdermal absorption of pentazocine [218]. It has been reported that the size of the pole head group is assumed to be the main factor for improving skin absorption. If the polar group is too small and has a suitable electrostatic charge distribution, the ceramides condense, resulting in good absorption. On the other hand, if the polar group of the surfactant is too large to be implanted in the polar areas of the ceramides, the drug will be poorly absorbed [211,216]. It is important to note that the stratum corneum and viable epidermis have been reported to have a gradient from lipophilic to hydrophilic. Accordingly, the selected surfactant should have an appropriate hydrophilic-lipophilic balance (HLB) value to improve penetration.

##### Phospholipids

Phospholipids are essential components of dermal formulations due to their natural origin and multifunctional properties. They serve as key components in some nanocarriers (such as SLNs and NLCs) and as surfactants in others (such as liposomes and niosomes). The three main classifications are monoacylphospholipids (lyso-phospholipids), saturated phospholipids, and unsaturated phospholipids. Monoacylphospholipids are commonly used as emulsifiers, especially in oil/water emulsions, or as flexibility modifiers of liposomes due to their detergent effect [219]. Saturated and unsaturated phospholipids, on the other hand, are generally used as key components of vesicular systems. Several types of egg yolk phosphatidylcholine liposomes have previously been studied for the transdermal penetration of retinoic acid. The authors found that liposomes containing egg yolk phosphatidylcholine had twice the skin penetration of a retinoic acid solution [220]. They concluded that the liquid or gel states of phospholipids are crucial for dermal delivery because they directly affect the fluidity of the vesicles formed. The use of gel-state phospholipids reduces the flexibility of the developed vesicles and their ability to penetrate the skin. It has been reported that unsaturated phospholipids are more suitable for increasing the dermal absorption of lipid nanosystems [219]. Perez-Cullell et al. investigated the impact of phospholipid type (saturated and unsaturated) on the transdermal delivery of sodium fluorescein. The authors showed that liposomes containing unsaturated phosphatidylcholine had superior skin penetration compared to those containing saturated hydrogenated phosphatidylcholine [221]. In addition to the phospholipid type, the phospholipid concentration can affect skin penetration. Carr et al. reported that liposomes with high concentrations of phospholipids penetrated the skin less than those with low concentrations of phospholipids [222].

Clarifying the factors that enhance or impede the skin penetration of nanosystems can aid in the design of an optimal lipid nanocarrier for drug delivery. Despite studies on the penetration of nanosystems into the skin, the mechanism and associated factors are not fully elucidated. It is known that the physicochemical properties of lipid nanosystems determine the interaction with biological systems and nanocarrier cell internalisation.

**Figure 4:**
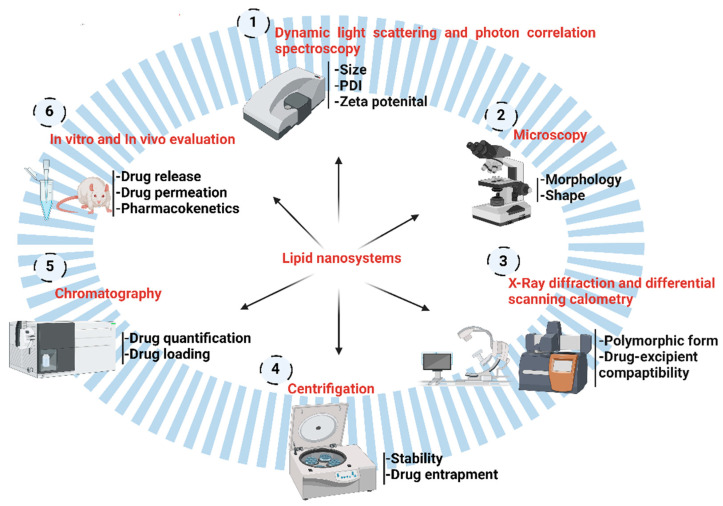
Common characterization and evaluation techniques of lipid nanosystems.

A summary of the different techniques used for the physicochemical characterisation and evaluation of lipid nanosystems is provided in Figure 4.

## 4. Methods for Assessing the Transdermal Flux of Nanoparticles

The application of drugs to the skin for medicinal or cosmetic purposes is among the earliest registers of traditional medicine. The intention to deliver potential candidates through the skin involves a principle of percutaneous absorption. The study of topical and transdermal absorption and the elucidation of this mechanism using the skin or skin substitutes have become indispensable. This section is devoted to the review of the different techniques used to study the skin absorption of nanoparticles.

### 4.1. In Vitro Methods

In vitro methods use rapid and cost-effective assessments of drug permeation through synthetic or biological membranes. The following subsections discuss the distinct types of diffusion cells, along with several types of membranes utilised.

#### 4.1.1. Diffusion Cells

Diffusion cells are the most popular in vitro screening tools for examining drug or nanoparticle permeation in the skin. It was first used by Dr. Thomas J. Franz in 1970. Diffusion cells usually consist of a donor chamber, a recipient chamber, and a membrane through which the drug enters [223].

#### 4.1.2. Franz Diffusion Cell

A Franz cell, also known as a thermostatic diffusion cell, is a common in vitro technique for studying the transcutaneous passage of compounds. There are two types of Franz diffusion cells: static and flow-through cell diffusion cells [224]. The receptor fluid is partially replaced during the experiment in the static diffusion cell. In flow-through diffusion cell, on the other hand, the circulating medium continuously fills the receiving fluid [225,226,227]. The cell consists of a donor compartment and a recipient container with an appropriate solution. The two compartments of the Franz cell are separated by a membrane, which can be artificial or made of a piece of skin (human or animal). The formulation to be tested is applied to the membrane in the donor compartment. The donor compartment can be closed (due to increased penetration values caused by high pressure) or open (measurement is based on atmospheric pressure). The kinetics of passage are determined by dosing the active substance at regular intervals in the receptor compartment. Sebe et al. [228] developed modified vertical diffusion cells for laboratory use. This modified diffusion cell has advantages such as a bubble-free environment in the receiving chamber when replenishing acceptor fluid, as well as ease of assembly and cleaning.

#### 4.1.3. Side-By-Side Diffusion Cell

A side-by-side diffusion cell is composed of donor and recipient compartments aligned horizontally, which allows them to have the same volumes and the same agitation efficiency. Because the skin is highly hydrated, this method is destructive to the membrane mounted between the compartments compared to other diffusion cells, resulting in an excessive permeation profile for the drug being tested [229].

#### 4.1.4. Jacketed Franz Diffusion Cell

**Figure 5:**
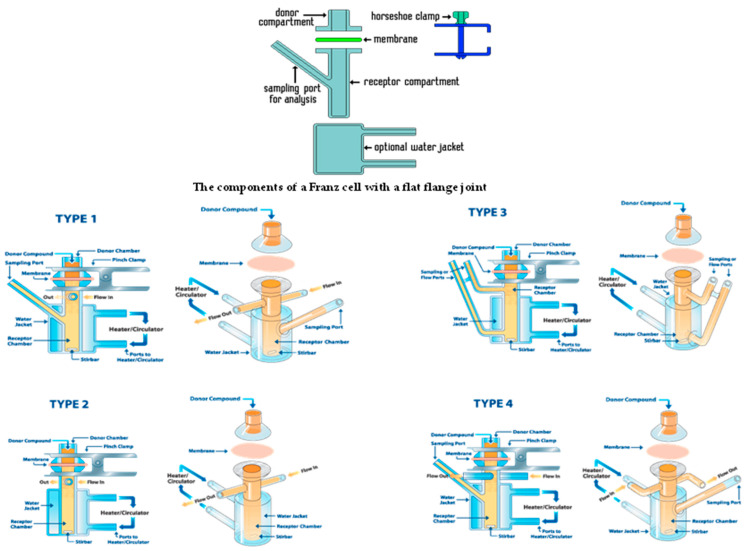
Illustration of the different types of diffusion cells used in the determination of transdermal flux. Images reproduced with permission from PermeGear, Inc.

It is a modified form of a Franz diffusion cell in which the diffusion cell has an outer chamber, i.e., a jacket containing water, which is generally used to maintain the diffusion cell at the desired temperature. It has superior permeation properties, including flow rate and permeability coefficient, as the temperature can be maintained when needed [229]. This device offers an innovative approach to testing drugs on real human skin and may help replace animal models [230]. Drug diffusion and heat transfer in the dermal layers of the skin can be predicted using mathematical modeling and computational fluid dynamics [231]. In general, this device consists of three main parts: a set of polymer-based microfluidic channels, a frame that surrounds the microfluidic channel system, and a sample holder that holds and inserts the membrane or skin sample into the membrane chamber [232]. Additionally, the skin-on-a-chip microfluidic platform enables cost-effective and reliable drug screening as the delivery setup is miniaturised [232]. Currently, a technology involving the connection of skin-on-a-chip devices to robotic systems for mid-throughput drug screening in the pharmaceutical industry is being validated and optimised [231]. The different types of diffusion cell are depicted in Figure 5.

### 4.2. In Vivo Methods

In vivo models provide a more accurate estimate of the amount of drug absorbed through the skin. These models include open-flow skin microperfusion, microdialysis, and direct assessment of pharmacokinetic and pharmacodynamic drug-plasma responses [233]. They provide more information about the effects of the drug on the human body than other methods [229].

### 4.3. Ex Vivo Methods

These methods included the tape stripping method, confocal Raman spectroscopy, confocal laser scanning microscopy and fluorescence lifetime imaging microscopy, all of which are discussed in the sections that follow.

#### 4.3.1. Tape Stripping Method

In this technique, the stratum corneum is removed by repeatedly applying tape to the surface of the skin, which causes the tape to partially remove the skin barrier. It has been widely used in dermatopharmacokinetics and drug penetration depth studies for topical and transdermal formulations [234].

#### 4.3.2. Confocal Raman Spectroscopy

The structure of the skin can be studied molecularly through spectroscopic methods. Raman spectroscopy is a promising spectroscopic technique based on the discovery of characteristic vibrational energy levels of a molecule excited by a laser beam that provides information about the molecular structure of tissue components without the use of fluorescent markers or dyes [235,236]. Accordingly, this method shows promise for detecting changes in the structure of skin components. It is also useful for tracking API penetration. Raman microscopy is now a powerful technique for fully understanding skin structure and percutaneous drug penetration [237,238].

#### 4.3.3. Confocal Laser Scanning Microscopy

Confocal laser scanning microscopy is used to track the transit of drugs through the transdermal route and has been widely used for a variety of applications, including the extent of drug penetration through the skin, the influence of different penetration enhancers and the interaction between the skin and drugs [229].

#### 4.3.4. Fluorescence Lifetime Imaging Microscopy (FLIM)

The method involves excitation with a high-frequency pulsed laser, detection of individual fluorescence photons, measurement of photon lifetimes emitted as a result of excitation light pulses, identification of the location of the laser point in the sample at the time of photon detection, and creation of a multidimensional distribution of photons based on these parameters [239,240,241,242]. FLIM can track the depth of nanoparticle penetration into the skin based on the varied fluorescence lifetimes of skin components and nanoparticles and their contribution to the multiexponential fluorescence decay function [243,244]. It is often used in combination with other techniques to study the structure of the skin. For example, the combination of FLIM and confocal microscopy provides the ability to directly measure epithelial morphology, biochemistry, and pathology in vivo.

**Figure 6:**
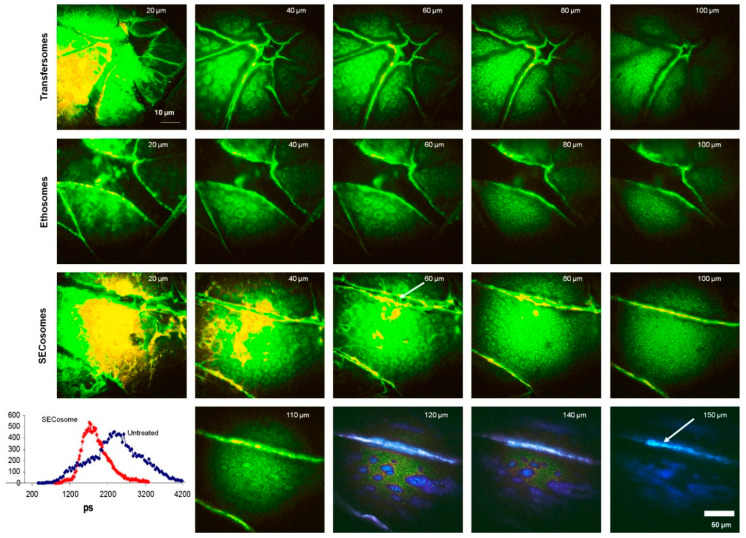
Penetration of 5-carboxyfluorescein-labeled siRNAs into flexible liposomes from freshly excised human skin. The sizes of the liposomes and the percent size decrease caused by filtration through a 30 nm porous filter were as follows: transferosomes 98 nm (30%); ethosomes 79 nm (11%); 58 nm secosomes (3%) and 98 nm rigid liposomes, (-). Reproduced without modification and with permission from [245].

Roberts et al. used [245] FLIM and multispectral imaging to study the transport of siRNA vesicles to viable epidermis after topical administration to excised skin. They found that rigid liposomes did not penetrate excised skin, but that penetration increased as the deformability of the liposomes increased as shown in Figure 6.

They also demonstrated that most penetration occurs through dermatoglyphs, with preliminary evidence that some of the more flexible liposomes transfer into deeper tissues and cells.

## 5. Application of Lipid Nanosystems for Skin Delivery Clinical Application of Lipid Nanosystems for Skin Delivery

The overall prevalence and pattern of skin conditions are influenced by the existence of contagious diseases and environmental conditions. Infections, eczema and dermatitis, pigmentation anomalies and acne are common skin diseases affecting communities [246]. To treat skin conditions successfully, the method of administration as well as the type of carrier vehicle used should be ideal. Topical application of drugs at the site minimises systemic effects and reduces the occurrence of side effects [139]. Topical and transdermal delivery of drugs present many advantages, such as patient compliance, avoidance of hepatic first-pass metabolism, and the possibility for self-administration [247]. The applications of lipid nanosystems for topical and transdermal drug delivery are discussed in the section that follows.

### 5.1. Topical Drug Delivery

#### 5.1.1. Infectious Diseases

Superficial and deep infections by bacteria, fungi and viruses cause various dermatological problems. Cellulitis, impetigo, and erysipelas account for common bacterial infections [248]. Group A streptococci and Staphylococcus aureus are the common causes of bacterial infections, and the former accounts for a substantial number of cases [246]. Dermatophytes, Candida and Malassezia species are common causes of superficial cutaneous infections, while herpes simplex virus is implicated in viral skin infections [248].

Difficult-to-treat bacterial infections of the skin are characterised by the ability of some pathogens to persist intracellularly, deep skin infections, and the development of antimicrobial-resistant bacteria. These challenges have necessitated the development of innovative approaches to the treatment of skin infections that involve the use of broad-spectrum bactericidal compounds loaded in specially tailored delivery carriers that effectively enhance the permeation of the drug molecule through the skin and cellular membranes [249,250,251].

A study explored the release of peptide human cathelicidin LL-37 from GMO-based cubosomes. LL-37 is a broad-spectrum bactericidal agent with immunomodulatory and wound healing properties; however, the peptide is prone to proteolytic degradation that limits its therapeutic use. The successful encapsulation of LL-37 in cubosomes resulted in delayed drug release and reduced susceptibility to enzymatic degradation [251].

Another study explored the use of ethosomes to enhance skin permeation of a conventional antimicrobial agent in treating deep skin bacterial infections. The efficiency of inhibiting infection by ethosomal erythromycin was compared to a hydroethanolic solution of erythromycin, and it was observed that the ethanolic solution was not effective in curing deep dermal infection. The action of the soft vesicles was thought to handle the efficient therapeutic effect [250]. The physicochemical characteristics of ethosomes enable them to overcome surface permeability barriers, as observed in bacitracin-loaded ethosomes that fuse with the outer membranes of fibroblasts and release the antimicrobial agent directly into the cell cytoplasm [249]. Therefore, lipid nanosystem can be used as delivery systems that overcome challenges of drug instability and ineffective drug concentrations at the site of action in the treatment of bacterial infections.

Cutaneous fungal infections affecting both the epidermis and dermis are treated with orally or topically administered drugs. Oral treatment of cutaneous fungal infections is associated with side effects and drug interactions, while poor drug retention in the skin and emergence of resistant strains are linked to conventional topical therapy. Thus, therapeutic alternatives such as the development of nanostructured drug delivery systems have been necessitated to address these challenges [252].

Improved antifungal activity was observed for ketoconazole loaded NLCs dispersed in Carbopol^®^ gel compared to conventional ketoconazole cream. Ketoconazole uptake in the skin with minimal systemic uptake and local irritation was reported for the formulated gel in comparison to the commercial product. The results of this study present NLCs as good candidates for controlling drug release and skin targeting [253].

Similarly, another study reported that SLNs significantly increased the accumulative uptake of miconazole in the skin in comparison to a marketed gel. Enhanced skin targeting was also observed for the miconazole loaded SLNs [254]. Prolonged drug release has been observed when SLNs were used as drug carriers. Prolonged release of miconazole nitrate has been achieved using SLNs dispersed a gel [255]. Other antifungal agents incorporated in lipid-based nanocarriers include butenafine [256,257] and amphotericin B [258].

Herpes simplex virus infection is commonly treated with acyclovir. Acyclovir has low oral bioavailability; consequently, it is administered in frequent and high doses for optimum therapeutic efficacy to be achieved. The high and frequent dosing of acyclovir is associated with unwarranted adverse effects and, therefore, the use of SLNs in overcoming these limitations has been studied [259]. Acyclovir-loaded SLNs showed controlled drug release and significantly higher accumulation of acyclovir in the stratum corneum, and dermal layer compared to simple acyclovir cream [260].

Controlled release, improved skin penetration, and enhanced efficacy have been observed when antimicrobial agents were incorporated into lipid nanosystems. This makes them a better choice, especially for preventing drug-resistant strains by providing effective therapeutic concentrations for a long time.

#### 5.1.2. Inflammatory Diseases

Psoriasis and atopic dermatitis are common inflammatory skin conditions characterised by red, well-demarcated plaques with a thick, silvery scale and dry, itchy eczematous skin lesions, respectively [248]. The presence of microbes or chemical substances in the skin can lead to the activation of different subgroups of T cells in the immune system. In normal skin, activation of these cells initiates an inflammatory reaction that minimises or increases chemical or microbiological threats. In inflammatory skin conditions caused by autoimmune processes, TH1 and TH2 cells show a misdirected response against the body itself. In allergic processes, Th2-mediated inflammatory responses, such as in allergic contact dermatitis, occur after chemical or environmental exposure. Autoimmune skin disorders and dermatitis are characterised by the absence of regulatory T cells that suppress immune responses and help to prevent autoimmunity and attenuate inflammation [261].

Underlying causes of inflammatory processes can be genetic, hormonal, or environmental. Skin disorders result from overloading of the defense system of the skin typified by increased proinflammatory mediators and reactive oxygen species. Accordingly, immune modulating and antioxidant agents are used to treat these inflammatory processes [261]. Topical application of anti-inflammatory agents has the advantage that high drug levels can be achieved at the site of disease and systemic side effects can be reduced compared to oral or parenteral drug administration. Glucocorticoids, retinoids, nonsteroidal anti-inflammatory drugs, and COX-2 inhibitors are among some of the therapeutic agents used to treat inflammatory skin conditions [20]. The use of lipid nanosystems as carriers for anti-inflammatory agents shows that they enhance percutaneous absorption and even allow drug targeting to the skin and its substructures, potentially improving the benefit-risk ratio of topical therapy [20].

Psoriasis is one of the most common skin disorders and is considered to have key genetic underpinnings. The skin condition is characterised by excessive growth and aberrant differentiation of keratinocytes, and the application of psoralens combined with long wavelength ultraviolet (UV) radiation (PUVA) as treatment shows slower cell replication and a good clearance rate in psoriasis patients [262]. Hyperproliferative psoriatic skin presents as a skin barrier for psoralen permeation; consequently, SLNs and NLCs are considered particularly useful for the administration of lipophilic psoralens [263]. NLCs have shown potential for exploitation as carriers with improved drug permeation for psoriasis therapies because of their solubility enhancement, small particle size, and occlusive effect. Enhanced psoralen permeation can be useful in improving the skin absorption of drugs, while sustained release is important for drugs with irritating effects at high concentrations or to supply the skin with drugs over a prolonged period [263].

Various corticosteroids have anti-inflammatory and antiproliferative effects and range from super potent, middle strength and less potent. Acute inflammatory skin lesions of the face and intertriginous arears are treated with low- to medium-potency agents, while chronic, hyperkeratotic, or lichenified lesions on the palms and soles are generally managed with highly potent agents. Corticosteroids are, however, associated with adverse effects that include atrophy, masking of infections, tachyphylaxis and hyper/hypopigmentation [264].

Halobetasol propionate (HP) is a highly potent corticosteroid used to treat eczema, psoriasis, dermatitis, allergies, and rash. The therapeutic application of HP is limited by its low aqueous solubility and side effects, including skin irritation, burning, pustulation, erythema skin atrophy and leukoderma. To minimise these undesirable effects, the development of suitable drug delivery systems that control and sustain release while selectively targeting psoriatic lesions to reduce irritancy and antiproliferative activity is appropriate [265]. Developed SLN dispersions with HP have shown less skin irritation, greater occlusivity and slower drug release than conventional gels and marketed products. The improved drug stability and encapsulation of HP in SLNs could potentially overcome the adverse effects of HP and offer efficacy in skin disease treatment and improved patient compliance [265].

Nonsteroidal anti-inflammatory drugs (NSAIDS) are applied topically to treat various kinds of pain and inflammatory conditions. NSAIDS are potent analgesics, antipyretics, and anti-inflammatory agents that are used in the treatment of osteoarthritis and rheumatoid arthritis. In an arthritic condition, a drug delivery system that exhibits controlled release for a prolonged period is required to satisfy the goals of the treatment, such as reducing pain and inflammation, slowing disease progression, and preventing adverse reactions [266].

To this effect, Patel et al. prepared NLC-based topical gel of the NSAID aceclofenac which showed significantly higher steady-state flux, permeability coefficient, and entrapment ratio when compared to the commercial product. Inhibition of inflammation was also observed to occur at a larger magnitude in the prepared NLC drug carriers than in the commercial product. Therefore, NLC carriers can be utilised to achieve faster onset and prolonged action of aceclofenac [266].

Ethosomes have been used to improve the transdermal delivery of flurbiprofen, an anti-inflammatory agent. The authors showed an increase in analgesic activity and reduced paw edema [267].

Cyclooxygenase 2 inhibitors (COX-2), e.g., etoricoxib, are another class of pharmacological agents used in the treatment of chronic inflammatory degenerative diseases such as rheumatoid arthritis and osteoarthritis. Topical application is preferred because systemic side effects are eliminated, patient compliance increases, first-pass metabolism is avoided, and drug levels are maintained [268]. The use of nanoemulsion [268,269] for etoricoxib both demonstrates prolonged drug release that is ideal in the treatment of chronic inflammatory skin conditions.

The application of SLNs as drug carriers can also be extended to natural products. Curcumin is a naturally occurring polyphenol with a variety of pharmacological effects, including anticancer, anti-inflammatory, antimicrobial, and antioxidant activities. Curcumin can potentially be used in the treatment of eczema, dermatitis, pigmentation, acne, psoriasis, and exfoliative skin disease [270]. However, the limited stability, solubility and poor permeability of polyphenols limit their suitability for dermal targeting; therefore, SLNs can be used to resolve this issue. A developed curcumin-SLN engrossed topical gel showed enhanced skin deposition, efficient occlusion properties, improved antioxidant activity and inhibition of tyrosinase enzyme activity compared to a conventional curcumin plain gel [270].

#### 5.1.3. Anti-Aging and Cosmetics

Cosmetic formulations are designed to provide additional nourishment to the skin and usually have aesthetic and personal hygiene functions. The disturbance in skin function caused by some systemic diseases, such as vitamin deficiencies or endocrine gland pathologies, may require the use of cosmetic formulations that contain pharmacologically active ingredients [80].

Lipid nanosystems reportedly exhibit some skin protective properties by way of occlusion, adhesion, and lubrication [271]. Due to their small size, lipid nanosystems form a monolayer film that retards the loss of moisture caused by evaporation due to their hydrophobic nature, and their hydrating properties enhance skin elasticity, making them ideal for use as antiaging products. The mechanical and lubricating effects of lipid nanosystems could potentially reduce the desire to scratch, which is particularly useful in reducing skin damage from irritation and allergic reactions [80,272]. In addition to the protective and pharmaceutical functions, the elegance and appearance of the product itself are key features to consider, SLNs and NLCs largely contribute to this because of their submicron size, pearl-like properties and protective action, which is a consequence of their lipid composition [83].

A study that evaluated the effect of an lipid nanocarrier loaded with tocopheryl acetate on the hydration, biomechanical properties and antioxidant ability of human skin showed that the lipid nanocarrier produced immediate and long-lasting relief from skin dryness. The study further assumed that the increased skin hydration was due to the presence of the nanocarrier rather than tocopheryl acetate [271].

Acne vulgaris is a common skin disease affecting almost 85% of people aged 12–25 years. The disease condition is characterised by 5α-reductase-I inducing dihydrotestosterone (DHT) formation that stimulates sebocyte proliferation and sebum production during adolescence [273]. Treatment options for acne vulgaris include hormonal therapy and 5α reductase inhibitors; however, oral administration is associated with side effects that include loss of libido gynecomastia, vasomotor flushing, and loss of bone mineral, particularly in males [273,274]. Consequently, topical application has been considered. Nanoparticle and microsphere systems loaded with joint cyproterone acetate (CPA)/ethinyl estradiol were studied, and it was observed that skin absorption of CPA was enhanced following SLN application [273].

Another study formulated adapalene-loaded SLNs for the treatment of acne. These AD-SLNs were further dispersed in a gel containing minocycline for topical application. The ex vivo permeation study through goat’s skin revealed gel exhibits high permeation of both drugs’ solutions. An in vitro antibacterial study indicates that the developed formulation has the same antibacterial activity as the marketed formulation [275].

Nayak et al. [276] co-loaded coenzyme Q10 and retinaldehyde into NLCs and studied the effectiveness of this formulation in reducing wrinkles. The formulation was incorporated into Carbopol^®^ 934P-NF gel. Clinical studies showed increased cellular uptake without cytotoxicity, while the in vitro release profile was prolonged.

Lipid nanosystems have shown improved drug permeation, prolonged drug release, reduced drug degradation, and improved therapeutic activity compared to conventional vehicles. Furthermore, the occlusive, hydrating and adherence effects on skin make them suitable bases for cosmetic products. The submicron size and pearl-like properties make them aesthetically pleasing to patients, potentially improving compliance. 

### 5.2. Transdermal Delivery

Lipid nanosystems have many advantages, overcoming the limitations of transdermal delivery and opening new possibilities. Deeper skin penetration can be achieved with lipid nanosystems, as they act as permeation enhancers by disrupting the skin barrier [184]. Additionally, these nanosystems can prolong drug release, significantly reducing dosing frequency and side effects. They can also transport hydrophilic and hydrophobic compounds as well as larger molecules. The improved pharmacological activity and efficacy should also be attributed to skin adhesion and the proximity of the nanosystems to the target cells [277,278,279]. The application of lipid nanosystems for transdermal drug delivery is discussed herein.

#### 5.2.1. Transdermal Delivery of Small Molecules

Lipid nanosystems have been used successfully as transdermal carriers for a range of drugs, including anti-HIV, NSAIDs, steroids and local anesthetics. Jain et al. used elastic liposomes to improve the systemic bioavailability and sustain the release of zidovu dine. The authors demonstrated that elastic liposomes enhanced the transdermal flux and improved the site specificity of zidovudine [280]. In another study, ethosomes were used to increase the transdermal flux of clotrimazole. The results showed an enhanced transdermal flux (56.25 µg/cm^2^/h) and a decreased lag time (0.9 h) [281]. A nanoemulsion was developed for the transdermal delivery of curcumin. The formulated nanoemulsion improved curcumin permeability and prevented the drug from chemical degradation [282]. Avanafil is used in the treatment of erectile dysfunction but is reported for its poor aqueous solubility. Karukula et al. used SLNs for the transdermal delivery of avanafil. The results revealed increased drug permeability [283].

Rostamkalaei et al. used ultrasonication to prepare metformin loaded SLNs nanogels with improved skin permeation. They showed that the amounts of metformin detected in the skin layers and the receptor chamber at all sampling times were higher for nanogel compared to metformin gel [284].

Chen et al. [285] develop a novel topical delivery system of carvedilol for skin cancer prevention using transferosome as the nanocarrier. Compared to the free drug, transferosome loaded carvedilol released through the dialysis membrane and permeated through the porcine ear skin at a slower rate, but similarly depositing the drug in the epidermis and dermis of the skin.

Kaur et al. [286] formulated nanoemulsions (NE) and hydrogel-based NE for transdermal delivery of fluvastatin. In vitro and ex vivo permeation studies showed significantly higher permeation of NE and NE gel in comparison to Fluvastatin solution, indicating that NE gel can effectively penetrate through skin layers. In vivo anti-osteoporotic results demonstrated the formation of new bone in the trabecular region of osteoporotic rat femurs through micro-CT scanning radiographs Recently, Altamimi et al. used elastic liposomes to enhance the transdermal delivery of lutein to control breast cancer. Compared to the drug suspension, the formulated elastic liposomes showed maximised cell inhibition [287].

#### 5.2.2. Transdermal Delivery of Peptides and Proteins

Cevc et al. used transferosomes to deliver insulin to the systemic circulation. Transferosome-loaded insulin composed of phosphatidylcholine and sodium cholate was formulated and compared to subcutaneous injection. Passive permeation of insulin through undamaged human or animal skin is very small due to its size (a molecular weight of ∼6000 Da) and polarity. However, the authors reported that radiolabeled insulin loaded in transferosomes permeated the skin and reduced blood glucose levels in mice. A single noninvasive, transdermal delivery of insulin in transferosomes has been shown to produce systemic normoglycemia lasting at least 16 h [288].

Similar results were reported by Guo et al. [289] using flexible vesicles composed of lecithin. The vesicles loaded with insulin were prepared by reverse-phase evaporation and treated further by sonication. The in vivo hypoglycemic study showed a decrease (21.42 ± 10.2% at 1 h) in blood glucose levels with flexible vesicles.

Foldvari and co-workers [290] evaluated the feasibility of delivering interferon (IFNα), an antiviral agent used in the treatment of condyloma acuminatum, into human skin using liposomes. The authors demonstrated that liposome encapsulation increased transdermal delivery of IFNα and provides a possibility for a more immediate clinical application [290].

#### 5.2.3. Transcutaneous Immunisation

Vaccination is one of the most effective strategies available in the continuous fight against infectious pathogens. Vaccination stimulates a targeted immune response and the development of persistent immunological memory, both of which serve to protect the recipient against future infections. Most vaccines are currently given by subcutaneous (SC) or intramuscular (IM) injections, which can be uncomfortable and require sterile procedures as well as skilled healthcare professionals [291]. As a result, this technique is associated with poor patient compliance, especially when dealing with children. In this context, transcutaneous administration (TCI) offers a realistic alternative to invasive routes of administration (SC and IM), as well as consistent blood levels, fewer overall adverse effects, and greater patient compliance. TCI is both cost effective and convenient.

TCI is a new vaccine delivery technique that involves applying an antigen with an adjuvant to the outer layer of the skin and then delivering it to the underlying Langerhans cells (LCs), which act as antigen-presenting cells [292,293]. Owing to the presence of numerous LCs under the epidermis, TCI triggered a robust immunological response. LCs are also associated with epidermal antigen-presenting cells and migrating T lymphocytes [294]. Before being presented at the cell surface for T-cell recognition, all antigens are first broken up into small segments that bind to MHC molecules. Transcutaneous vaccination induces robust cell and mucosal (IgA, IgG) responses [293]. Antigen-specific CD4+ (helper) and CD8+ T-cell responses are strongly activated. Dermal tissue, in fact, has more antigen-presenting cells than muscle or SC tissue. All these cells form the skin’s immune system, which is known as skin-associated lymphoid tissue (SALT). SALT in connection with lymph nodes may cause a cell-mediated and humoral immunological response [295]. However, the impermeable characteristic of the stratum corneum represents a significant barrier to dermal vaccine delivery. Advances in elastic liposomes (especially transferosomes and ethosomes) for various diseases have created a unique opportunity for TCI.

When compared to other vaccinations, the delivery of the hepatitis B surface antigen using ultra deformable liposomes exhibited higher levels of both cellular and humoral immune responses [296].

In another study, spectral bioimaging analysis, flow cytometry and spectral bioimaging analysis were used to provide evidence that hepatitis B surface antigen-loaded ethosomes were efficiently internalised by dendritic cells and were able to induce an immune response that was primarily of the TH1 type [297].

Another study compared the immunogenicity of the carboxyl-terminal 19 kDa fragment of merozoite surface protein-1 of *Plasmodium falciparum* when administered intramuscularly and subcutaneously through elastic liposomes. The results showed enhanced transcutaneous immunisation with elastic liposomes, as well as strong and long-lasting specific IgG responses and cytophilic isotype antibodies [298].

Transcutaneous immunostimulants with an Ag 85a deformable vesicle (immunodominant antigens of Mycobacterium tuberculosis, which causes tuberculosis) stimulate and recruit more CD11c-positive cells to the draining murine lymph nodes and generally stimulate immune cells than immunogen injections [299]. Transcutaneous immunisation also provides better protection to mice against tuberculosis infection.

Compared to ointment formulations, the encapsulation of outer membrane antigens of Salmonella enterica into a nanoemulsion showed greater epidermal and transfollicular antigen uptake and resulted in much higher IgG production [300].

### 5.3. Gene Delivery

There are two fundamental methods for therapeutic gene delivery via the skin: ex vivo and in vivo gene delivery [183]. Ex vivo gene delivery requires a skin biopsy to extract cells outside for growth and gene insertion in the culture, followed by a skin graft. In contrast, in vivo gene delivery includes the direct transfer of genetic material to the patient’s undamaged skin tissue and is hence a simple and more direct method. Compared to viral vectors, nonviral vectors demonstrated biosafety, reduced pathogenicity, low cost, and ease of production [183,301]. In this regard, lipid-based nanosystems are an innovative alternative for transdermal gene delivery. Various lipid-based nanosystems have been used for transdermal gene delivery. However, the literature reports that elastic liposomes are mostly used for the delivery of genes. This can be explained by their ultradeformability, absence of cholesterol, and capability to permeate across microscopic pores of skin [287].

Manosroi et al. loaded [302] tyrosine kinase plasmid (pMEL34) in elastic cationic niosomes prepared by the chloroform film method with sonication and rehydrated with 25% ethanol. Compared to the free plasmid, pMEL34 loaded into elastic cationic niosomes demonstrated the highest tyrosinase gene expression and tyrosinase activity. The authors suggested the potential use of elastic niosomes as a transdermal delivery system for the tyrosinase gene in vitiligo therapy.

In another study, the effect of the type of edge activators on the formulation and transdermal delivery of plasmid DNA-loaded ultra-deformable liposomes was reported. The results demonstrated that liposomes containing bile salt edge enhancers (sodium deoxycholate and sodium cholate) are more suitable for the transdermal delivery of plasmid DNA [303]. Other promising reported results for liposomes include [303,304].

## 6. Regulatory Aspects

The rapid growth of nanotechnology has enabled innovation in a wide range of industrial fields, including drug delivery systems. Although many other applications are still in the research and development phase, some are now commercially available [185]. Increased drug solubility, better bioavailability, and targeting possibility are just some expected benefits of nanosystems. However, due to their characteristics and potential large-scale use and exposure, these systems may represent a danger to human health and the environment. Well-defined global guidelines could minimise the uncertainties and lack of understanding of the impacts of lipid nanosystems on health and safety issues. This will ensure manufacturing transparency and increase public acceptability.

There are attempts worldwide to regulate the manufacture of nanosystems via legislation or guidelines and recommendations [305]. Currently, there is no legislation exclusively devoted to the regulation of nanosystems in any country [306,307,308]. Current FDA guidelines are considered by many countries to be adequate and sufficiently detailed to control lipid nanosystems [309,310]; however, revisions have been recommended by many stakeholders, including the European Parliament [311] and nongovernmental organisations (NGOs) [312]. Several expert organisations, including EU scientific committees and agencies, the Organisation for Economic Co-operation and Development (OECD), the International Organisation for Standardisation (ISO) and the Food and Drug Administration (FDA), are engaged in this sector and note the need for additional guidance to assess possible risks and suggestions to ensure safe use of lipid nanosystems. Relevant regulatory issues for lipid nanosystems include a clear definition of the term “nanosystem”, specific information requirements to assess the risk, authorisation procedures and provisions to ensure the traceability and transparency of clinical use, such as notifying clinicians for products containing lipid nanosystems [313,314].

Common regulatory guidelines include identifying nanosystems based on their size, chemical composition, surface properties, and stability. These characteristics are very important for identifying potential interactions and estimating how long they may persist in the body, providing information for risk assessment. Many analytical techniques accompanied by imaging methods are commonly used to assess lipid nanosystems. In addition, in vitro and in vivo studies provide important information about their behaviour in the body.

The success of any formulation relies on the large-scale translation bench. Such translation faces major challenges due to product stability and batch-to-batch variability, which can drastically alter formulation characteristics and, therefore, impair therapeutic success [315]. Large-scale production must be carried out in an environment that complies with Good Manufacturing Practice (GMP) requirements, which are based on rigorous and robust procedures, qualified equipment, and well-trained personnel. However, setting up an environment that complies with GMP requirements requires a significant financial investment.

In terms of safety, the regulatory status regarding the toxicity of excipients is critical for the formulation of a marketed lipid nanosystem. It is important to note that all components are not inert; in fact, some may be harmful in high amounts. The components to be used must come from the EMA and FDA lists of excipients, known as "generally regarded as safe" (GRAS). These lists are updated regularly to consider those that are newly approved [185,316]

## 7. Future Prospects

While it has been successfully demonstrated that the use of lipid nanosystems for dermal and transdermal applications is desirable and ultimately capable of achieving improved outcomes, there remains a need to understand that their use is not a one size fits all approach and, in some cases, may require additive manufacturing for the successful achievement of treatment outcomes.

To this effect it is worth considering the development of additive technologies that can exploit advantages of multiple technologies and these include loading these nanomaterials in nanofibers [317,318], hydrogels [319,320], or microneedles [321,322] for improved treatment outcomes. Some of the strategies are discussed herein.

### 7.1. Stimuli Responsive Nanocomposite Gels

The use of additive carriers to administer the nanocarriers presents an opportunity to derive further utility for these technologies. Aqueous suspensions of lipid nanocarriers could potentially run over the application site and may not be returned long enough to have substantial effect. The use of stimuli-responsive carriers could overcome such challenges. Some of the stimuli that can be utilised for transdermal and dermal delivery include temperature, pH and ionic species concentration.

Thermosensitive hydrogel systems offer the chance to administer a liquid solution or suspension containing a payload that, after administration, when a particular target temperature is reached, forms an in-situ gel at the site of administration. These systems consist of injectable fluids that can be introduced into the body with a minimum amount of trauma before gelling in the targeted organ, tissue, or body orifice. When compared to delivery technologies that are already pre-shaped in their final form before insertion, these gelling systems offer a number of advantages. For instance, if non-biodegradable materials are used, injectable fluids do not require surgical interventions for implantation and/or removal. In transdermal applications, these systems can form a film on the applied area and provide enhanced drug retention. Moreover, straightforward procedures like blending can be used to combine various therapeutic agents [323,324,325,326,327].

### 7.2. Ionic Liquids

Ionic liquids (IL) are salts that are entirely composed of ions and have a melting point below 100 °C [328,329]. The production of IL from API salts has a long history, as liquid formulations avoid polymorphism found in crystalline forms and promote dissolution into water, resulting in higher bioavailability [328]. In recent years, there has been an urgent need for novel scientific advances that result in pioneering and efficient drug therapies. Traditional strategies are currently being pursued to the point where it is difficult to develop new chemical entities that are effective. It is estimated that less than 10% of API currently being evaluated in clinical trials will be approved for market use. This significantly reduces the availability of effective medications for those in need [330]. Approximately half of all existing API are given as salts. It is possible to finely modify the physicochemical and biopharmaceutical properties of a given API by pairing various counterions. Melting temperature and solubility are important parameters in pharmaceutics because they affect drug processing and bioavailability [331].

Utilising these liquids as alternatives to water as the dispersant of nanocarriers could improve outcomes with regards to hydrolysis and oxidation of lipid components. It has further been explored to use ionic liquids as hydrating medium and has been suggested that the glass transition temperature of lipids could be lower in ionic liquids requiring a lower input of energy to form liposomes and other vesicular systems. The use of ionic liquids could yet be the next frontier in improving outcomes of lipid nanocarrier drug delivery in transdermal and dermal applications [332,333,334].

### 7.3. Deep Eutectic Solvents

Deep eutectic solvents (DES) are a class of solvents that are relatively new. They are created by combining organic compounds to form a eutectic mixture. The eutectic mixture has a melting point that is significantly lower than the sum of its parts [130]. However, DES is also used to describe a variety of liquids that form at non-eutectic ratios. As they get around some of drawbacks associated with IL, DES have been looked at as potential alternatives to traditional IL. As a consequence of their biodegradability, low toxicity profile, and that they can dissolve a variety of different chemical compounds, natural DES, which is composed of components of natural origin, are particularly promising as solvents and dispersants for pharmaceutical use.

API has been incorporated into microemulsions in DES and DES/water mixtures as a transdermal drug delivery system [335]. Compounds dissolved in DES are also known to improve skin penetration [336,337]. To prevent polymorphism in solid-phase mixtures, DES can also be created when one or both components are themselves API [338].

Vesicle formation in DES has been reported for a number of phospholipids [339,340,341] and glycolipids in the context of bilayer drug delivery. When compared to lipids in vesicles in water, the chain melting temperature of lipids is typically higher in DES. However, it also depends on the hydrogen bond donors of the solvent [340].

Utilisation of these solvents has been shown and can be considered a new avenue to improve transdermal and dermal applications for lipidic nanocarrier drug delivery.

### 7.4. Nanofiber Technology

Nanoscale polymers have a wide range of applications, particularly in medical applications, where the electrospinning process is one of the most convenient ways to directly and continuously prepare nanofiber membranes [342,343,344]. The main benefit of this technique is the variety of drug release mechanisms that can be tailored for use. It has been reported that the mechanism of API release from nanocarrier loaded nanofibers could be due to drug release from the nanocarrier and subsequent diffusion through the nanofiber membrane or the nanoparticle diffusing through the nanofiber wall and releasing API [345].

This technique could be used to develop biphasic drug release and be utilised in multi-drug release mechanisms.

### 7.5. Microneedle Technology

Microneedles (MN) are a collection of micro-scale needles that can penetrate the outermost skin layer, known as the stratum corneum, and enter the skin to achieve minimally invasive transdermal drug delivery [346].

MN technology allows for the administration of nanocarriers directly into the skin while not stimulating the nerve endings. It is an efficient route of delivering nanocarriers that may not have sufficient penetration across the stratum corneum. It has been demonstrated to have efficiency in the delivery of elastic liposomes [322] and NLC [347]. The use of this technology could yet provide another future breakthrough in long delivery of lipid nanocarrier vaccines and drugs.

A summary of potential future applications of additive manufacturing is provided in Table 3.

## 8. Conclusions

The utilisation of advanced nano drug delivery systems has been the driving force to development of novel medicines due to their biocompatibility, biodegradability, target specificity, and lower dose frequency. The use of lipid nanosystems has long been proven to be an innovative technology capable of circumventing many of the challenges associated with conventional drug delivery. They have exhibited flexibility in routes of administration and have been able to improve treatment outcomes in many different disease classes.

The utilisation of lipid nanosystems in dermal and transdermal applications is widely regarded as the next frontier in the treatment of topical and systemic diseases due to the ability of the nanomaterials to achieve desirable spatiotemporal delivery of payload.

Despite the many successes of lipid nanosystems in dermal and transdermal applications, it remains to be seen whether their utilisation will ultimately improve the treatment outcomes of many diseases in the present and future diseases.

## Figures and Tables

**Table 1:** A non-exhaustive list of marketed transdermal systems.

Active Pharmaceutical Ingredient	Brand Name	Manufacturer	Indication/Marketed Country
Rotigotine	Neupro^®^	Schwarz	Parkinson’s disease restless legs syndrome/USA
Isosorbide dinitrate	Frandor Tape^®^	Yamanouchi	Myocardial ischemia/Japan
Methylphenidate	Daytrana^®^	Noven	Attention deficit hyperactivity disorder/USA
Tulobuterol	Hokunalin^®^ Tape	Abbott Japan	Asthma/Japan
Fentanyl	Duragesic^®^	Alza/Janssen	Analgesic/USA
Scopolamine	Transderm-Scop^®^	Alza/Ciba-Geigy	Motion sickness/USA
Ethinyl estradiol	Ortho Evra^®^	Ortho-McNeil	Contraception/USA
Clonidine	Catapres-TTS^®^	Boehringer Ingelheim	Hypertension/USA
Nicotine	Nicotinell^®^	Ciba-Geigy	Smoking cessation/Switzerland
Nitroglycerin	Transderm-Nitro^®^	Ciba-Geigy	Stenocardia/USA
Testosterone	Androderm^®^	Watson Pharma	Testosterone deficiency/USA

**Table 2:** A brief description of conventional liposomes and particulate carrier systems.

Nanosystems	Basic Composition	Methods of Preparation	Benefits	Limitations	Refs
Nanoemulsions/Self-emulsifying systems	Oil, surfactant and co-surfactant/cosolvent	High and Low-energy emulsification	- High potential to increase the permeation of drugs through the skin. - Uniform deposition due to lower surface and interfacial tension - Thermodynamic stability	- Require large amounts of surfactants. - Limited information about the process of emulsification	[140,141,142,143]
Solid-lipid nanoparticles (SLNs)	Solid lipid, surfactant and cosurfactant	Emulsification-solvent diffusion, High-pressure homogenisation, Double emulsion technique	- Narrow size offers targeted and controlled drug delivery. - Enhanced bioavailability and chemical stability of both hydrophobic and hydrophilic drugs - Biocompatibility	- Lower drug loading capacity and drug expulsion during phase transition or lipid crystallisation - Unpredictable gelation tendency	[77,144,145,146]
Nanostructured lipid carrier (NLCs)	Blend of solid and liquid lipids, surfactant, cosurfactant	High-pressure homogenisation and ultrasound	- High drug loading capacity - Reduced risk of drug release during storage	- Irritation observed due to some surfactants.	[147,148,149,150]
Lipid nanocapsules (LNCs)	Oil, nonionic surfactant, lipophile surfactant	Phase inversion temperature technique	- Biocompatibility - Biodegradability - Targeted delivery	- Reduce stability over time. - Long formulation procedure	[85,151,152,153]
Liposomes	Cholesterol, phospholipids, antioxidants	Film hydration, solvent injection, and reversed-phase evaporation	- High biocompatibility - High targeting ability - Improved drug permeation	- Reduced skin penetration and less stable. - Low drug loading capacity	[89,154,155,156]
Ethosomes	Cholesterol, phospholipids, antioxidants, Ethanol	Film hydration, solvent injection, and reversed-phase evaporation	- Suitable for delivering lipophilic and hydrophilic drugs. - Optimal encapsulation efficiency as opposed to liposomes. - Cost-effective and simple method of preparation. - High deformability and elasticity with deeper skin penetration in comparison to liposomes	- High skin irritation due to high ethanol concentration.	[61,123,157,158]
Niosomes	Non-ionic surfactants, Cholesterol, Charge inducers	Film hydration, solvent injection, and reversed-phase evaporation	- More stable in comparison to liposomes. - Niosomes do not require any specialized handling and storage conditions. - Osmotically active. - These particles can entrap drugs with a broad solubility range. - Niosomes serve as a depot system for sustained drug release when there is a need. - The design is more flexible in terms of structure in comparison to liposomes. - Niosomes lead to improved topical, oral, as well as parenteral drug bioavailability. - Therapeutic efficiency of the entrapped drug is improved through limiting impact with target cells while minimizing drug clearance.	- Hydrolysis of encapsulated drug which reduces the shelf-life of the dispersion.	[159,160,161,162]
Transferosomes	Cholesterol, phospholipids, antioxidants, edge-activators	Film hydration, solvent injection, and reversed-phase evaporation	- Ultradeformable - Highly efficient entrapment of both small and high molecular weight drugs. - Effectively utilized in systematic and topical drug delivery. - Depot drug delivery is a possibility. - Protection encapsulated drug from metabolic enzymatic degradation.	- Chemically unstable. - Expensive and costly. - Phospholipid purity is a concern	[163,164,165]
Cubosomes	Glyceryl monooleate, poloxamer	Top down and bottom down techniques	- Ability for targeted release in addition to controlled drug release. - Amphiphilic, hydrophobic, and hydrophilic drugs are very easily encapsulated. - Easy preparation of the skin. - Biodegradable. - High bio-adhesive nature is exhibited by the cubic phases and are thus more convenient for the mucosal and topical drug delivery system.	- Expensive and costly. - Limited yield.	[166,167,168,169,170,171]
Glycerosomes	Phospholipids, glycerol, cholesterol	Film hydration, Solvent spherule, small unilamillar vesicles sonication	- Improved entrapment, fluidity, and stability - Nontoxic for transdermal delivery	- Glycerol leads to increase particle size and reduced drug release	[172,173,174]
Invasomes	Phospholipids, terpenes, ethanol, water	Mechanical dispersion, film hydration	- Possibility of encapsulating hydrophobic and hydrophilic compounds - Biocompatibility	- Leakage and fusion of encapsulated compounds - Less stable	[166,167,168,169,170,171]

**Table 3:** Summary of potential additive technologies and their associated outcomes.

Additive Approach	Benefits	Limitations	Refs
Stimuli-responsive nanocomposite gels	- Being able to tailor the carrier gels to respond to specific stimuli allows for modulated drug release reducing dose dumping and potential toxic effects. - Sustained release drug delivery is highly achievable.	- Additional Cost of production - Possibility of nanocarrier fusion	[324,325,326,327]
Ionic Liquids	- Improved penetration nanocarrier - Further protection of the vesicles against oxidation	- Increased risk of toxicity	[332,333,334]
Deep Eutectic Solvents	- Biodegradable - Possess a low toxicity profile. - Further protection of the vesicles against hydrolysis and oxidation	- Multi-step process - Potential interactions between carrier materials and DES.	[335,336,337,340,341]
Nanofibers	- Increased residence time - Applications in wound healing	- Requires specialised equipment. - Electric current could destroy carriers	[317,318]
Microneedles	- Increased residence time - Drug Release can be modulated. - Can load a large amount of nanocarriers.	- Multi-step process - Expensive	[321,322]

## Data Availability

Not applicable.
